# Localized Choroidal Melanoma Managed by Pars Plana Endoresection, Endodiathermy, and Endolaser

**DOI:** 10.7759/cureus.18077

**Published:** 2021-09-18

**Authors:** Ahmed J Naser, Maryam Alkhayat, Abdulhameed H Mahmood

**Affiliations:** 1 Ophthalmology, Salmaniya Medical Complex, Manama, BHR; 2 Ophthalmology, Prince Sultan Military Medical City, Riyadh, SAU

**Keywords:** transretinal endoresection, endodiathermy, endolaser, pars plana, choroidal melanoma

## Abstract

Choroidal melanoma, although rare, is the most common primary intraocular malignancy among adults, with an incidence ranging from 6 to 7.5 cases per million per year globally. This tumor originates from the uveal tract’s melanocytes in the eye. Melanomas usually arise from the sixth decade of age with increasing incidence with progressive age.

A 60-year-old male presented with a right-sided sudden painless decrease in vision due to a grade 4 vitreous hemorrhage. B-scan ultrasonography and CT scan of the orbits revealed a mushroom-shaped choroidal mass highly suggestive of choroidal melanoma, which was managed surgically with pars plana endoresection, endodiathermy, and endolaser.

Endoresection can be an effective method for the management of solitary non-metastasized choroidal tumors, especially those that have become symptomatic. Pre and post-surgical imaging are necessary, along with careful follow-up to detect any recurrence.

## Introduction

Uveal melanoma, although rare, is the most commonly diagnosed primary intraocular malignancy in adults with a mean age-adjusted incidence of 5.1 cases per million per year in the United States (from 1973 to 2008) [1,2]. These tumors, which represent 3%-5% of all melanomas, arise most commonly from choroidal melanocytes (90%) but can also arise from the iris (5%) and ciliary body (5%) [3,4]. The median age of diagnosis is approximately 62 years, with a higher incidence in males and a significantly higher incidence among Caucasians and those with fair skin [3,5,6].

Blurred vision is the most common presenting symptom of primary uveal melanoma [4], which was also the presenting symptom of our patient. Other symptoms include photopsia, floaters, and visual field loss, while almost one-third of patients remain asymptomatic until the time of diagnosis [3,4].

These tumors have a high propensity to metastasize, as 50% of patients develop metastatic disease despite primary therapy and active surveillance [3,6,7]. Liver is the most common site of metastasis, followed by lungs, bone, skin, and lymph nodes [6,8]. Once metastasized, uveal melanomas’ mortality significantly increases, as survival rates decline from around 80% at five years for non-metastatic tumors, to 15% at one year for patients with metastases [8,9], with reported median survival ranging from 4 to 15 months after the time of diagnosis of metastatic disease [10]. Therefore, a great emphasis has always been placed on early diagnosis and early primary treatment and avoiding unnecessary delays in the treatment of such cases, with special attention to continuous post-treatment surveillance [4,11].

The primary treatment for localized uveal melanoma can be classified into globe preserving therapy or enucleation, the latter being most appropriate for tumors that have caused permanent vision loss, those with extensive extraocular growth, or those with very large diameters [12]. Although enucleation remains the most common surgery performed for these cases, globe preserving therapies such as radiation therapy and laser, and less aggressive surgical approaches like transscleral resection and transretinal endoresection have all been gaining popularity as alternative therapies for patients seeking eye-retaining therapies [12-14].

Endoresection via the pars plana route (transretinal endoresection), with or without brachytherapy, has been described previously by several authors as an alternative to enucleation for posteriorly located uveal melanomas, with excellent local control and eye salvage rate, and was not associated with a higher risk of metastasis, death, or local recurrence than other reported techniques used to treat similar melanomas [15,16].

## Case presentation

A 60-year-old gentleman who was previously diagnosed with type 2 diabetes mellitus, hypertension, and a cerebrovascular accident, which he suffered two years prior to presentation, which left him with residual right-sided upper and lower limbs weakness, for which he is undergoing physiotherapy with continuous improvement, presented with a history of right eye sudden painless drop in the vision of one-day duration. He denied any history of trauma to the eye or head and gave no history of previous eye symptoms or surgeries. Apart from the right-sided upper and lower limb weakness, his general and systemic examination was unremarkable.

His ophthalmic examination showed visual acuity of hand movement in the right eye and 6/6 in the left eye, with normal intraocular pressure in both eyes (18 mmHg in the right and 12 mmHg in the left). Slit-lamp biomicroscopy showed grossly normal anterior segments of both eyes with immature cataracts. Fundus examination of the right eye by binocular indirect ophthalmoscopy showed no view of the retina due to a grade 4 vitreous hemorrhage, while fundus examination of the left eye was unremarkable.

**Figure 1:**
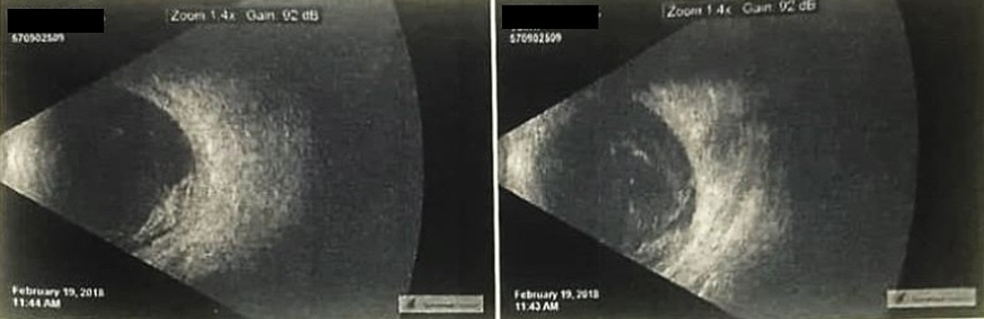
B-scan ultrasonogram of the right eye showing a mushroom-shaped mass protruding through the retina into the vitreous cavity (left). A shallow exudative detachment is seen at the edge of the mass (right).

**Figure 2:**
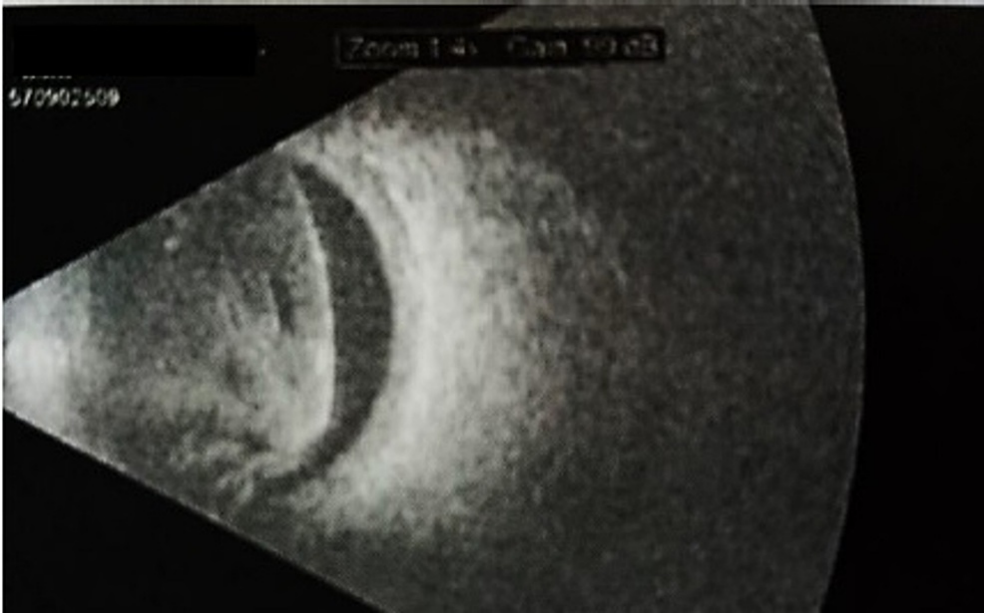
Another B-scan ultrasonogram of the right eye better demonstrating the exudative retinal detachment and the vitreous hemorrhage associated with the previously seen mass.

B-scan ultrasonography of the right eye showed a large mushroom-shaped mass invading the choroid and retina, and protruding into the vitreous cavity in the inferior aspect of the posterior pole (Figure 1) with a shallow exudative retinal detachment observed in the peripheral rim of the mass, with dense vitreous hemorrhage (Figure 2).

**Figure 3:**
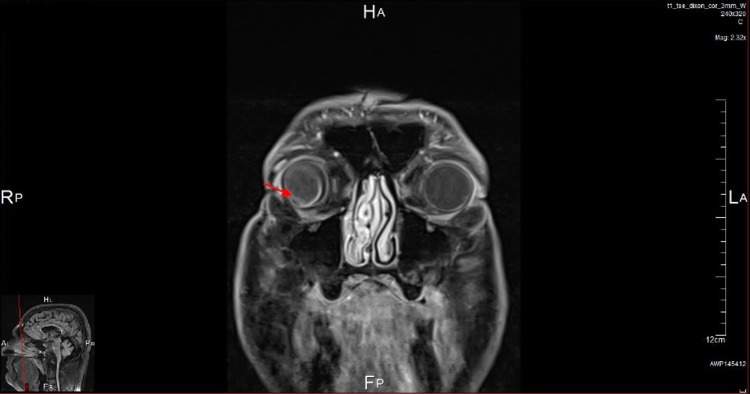
A coronal T1-weighted MRI scan of the orbits showing a homogenous moderately hyperintense mass within the vitreous of the right eye (red arrow) with associated retinal detachment.

**Figure 4:**
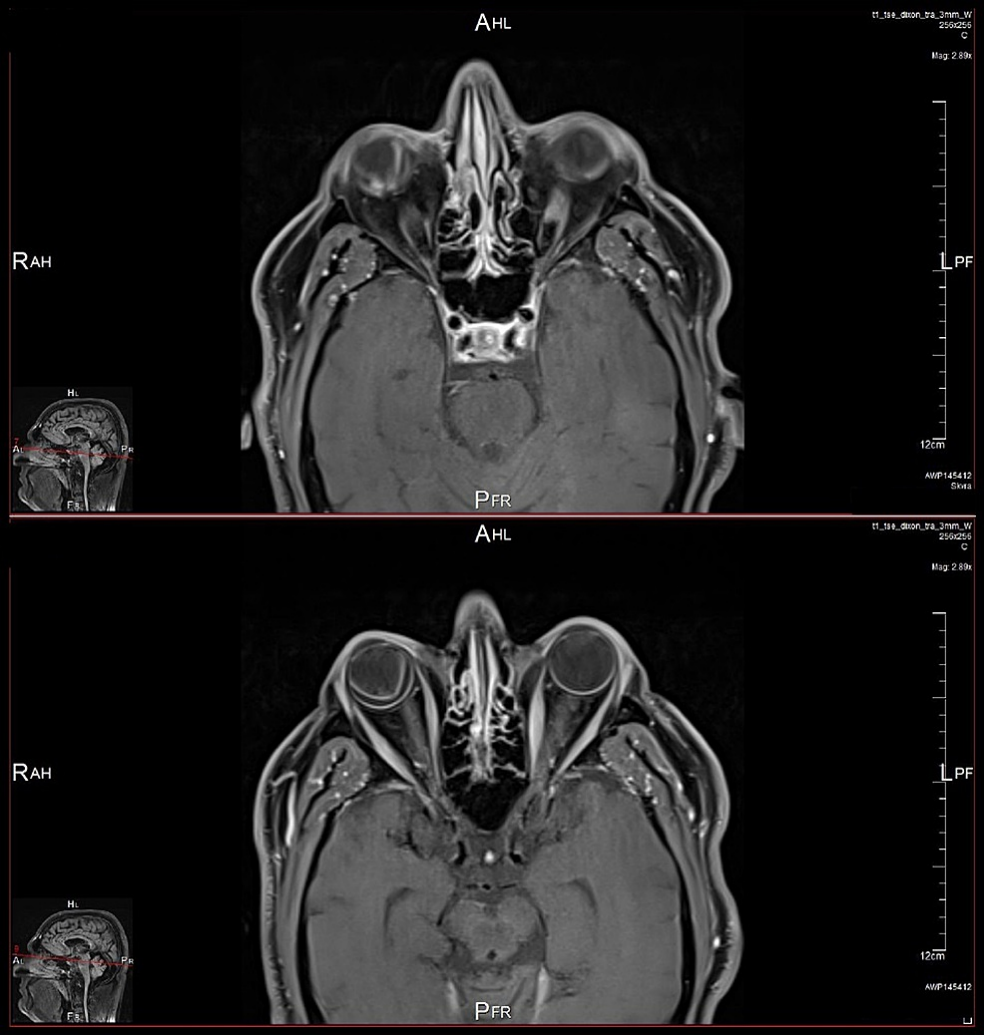
Two post-contrast axial T1-weighted fat-suppressed MRI scans of the orbits showing mild enhancement of the choroidal mass in the right eye (up) and the extent of the exudative retinal detachment of the same eye (down).

**Figure 5:**
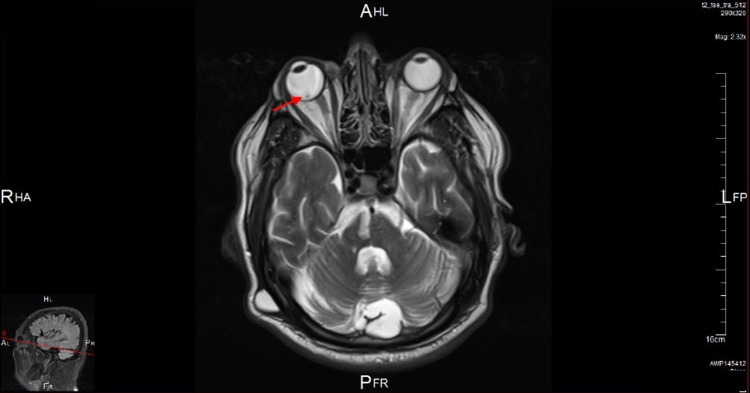
An axial T2-weighted MRI scan of the orbits showing a low-intensity small choroidal mass in the right eye (red arrow).

Magnetic resonance imaging (MRI) of the orbits was also done and showed a small mass suggestive of a choroidal melanoma along the posterior aspect of the right globe extending into the vitreous and measuring 8 x 6 mm. The lesion appears of high signal intensity on T1-weighted images and low intensity on T2-weighted images and shows mild contrast enhancement in the post-contrast images. The vitreous body shows an abnormal V-shaped appearance with abnormal high signal intensity on T2-weighted images mostly suggestive of vitreous hemorrhage with posterior vitreous detachment (Figures 3-5).

Additional testing included positron emission tomography (PET) scan, chest X-ray, and laboratory studies, which included complete blood count (CBC), serum electrolytes, and liver function test (LFT), which were all within normal limits, excluding the possibility of metastasis to the lungs and liver, and confirming that the choroidal mass is a localized primary ocular mass.

After discussing the treatment options with the patient, and due to the unavailability of plaque brachytherapy and the concurrent presence of a vitreous hemorrhage, which would already mandate a pars-plana vitrectomy, a decision was made to book the patient for an early surgical transretinal endoresection via the pars plana route.

**Figure 6:**
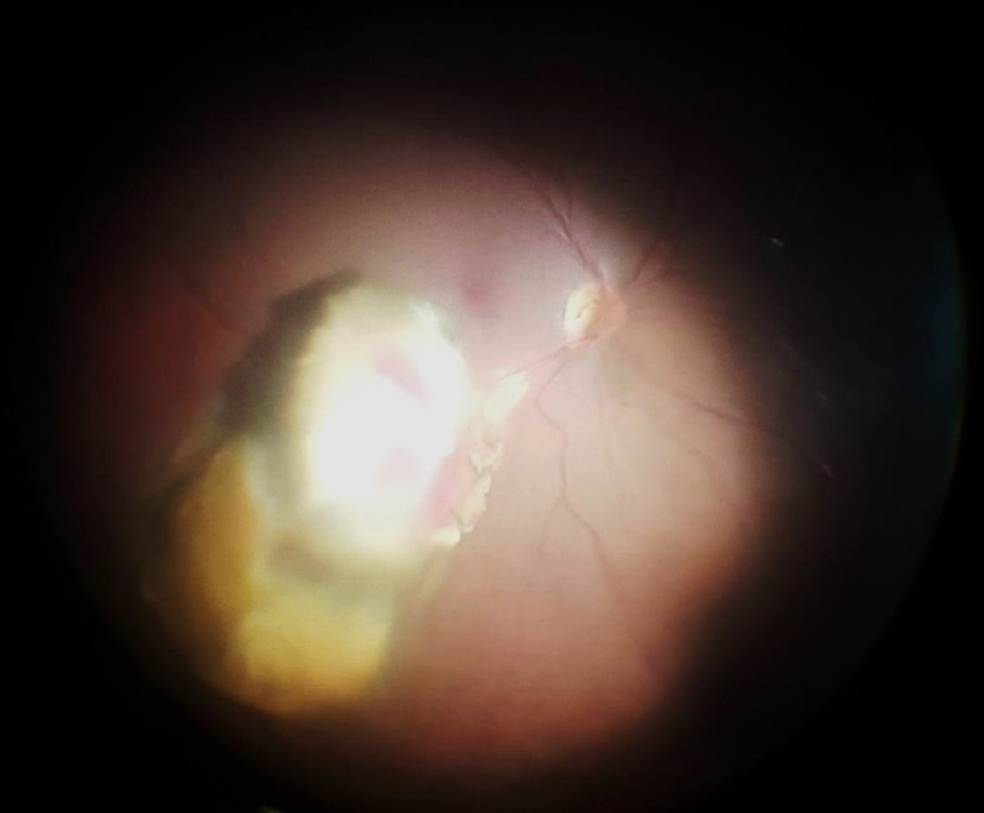
Intraoperative fundus photo of the right eye after completion of pars plana vitrectomy and clearing the vitreous hemorrhage, clearly demonstrating the size of the lesion and its proximity to the optic disc and macula.

**Figure 7:**
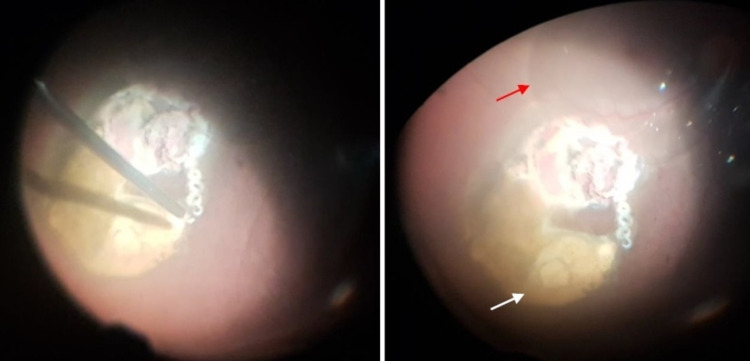
After complete endoresection of the mass using micro scissors and vitrector, an endodiathermy probe was used to cauterize the border of the resultant retinotomy (left). The edge of the perfluorocarbon liquid bubble used to protect the macula can be seen (right - red arrow) and the bare sclera underneath the resected mass can also be seen (right - white arrow).

**Figure 8:**
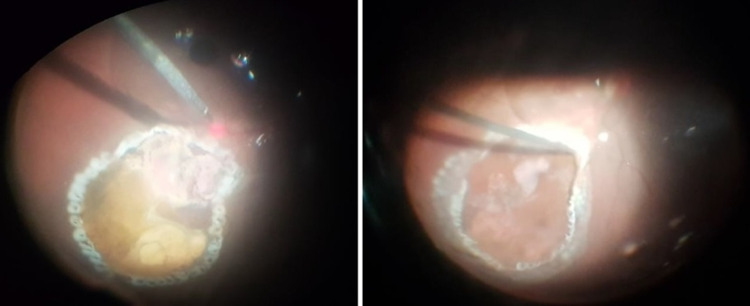
After completion of endodiathermy, a curved-tip laser probe was used to carefully perform argon laser photocoagulation, while the perfluorocarbon liquid can still be seen in place (left). Three rows of photocoagulation burns were applied around the resultant retinotomy, with the most posterior burns being more than one disc diameter from the fovea (right).

Under local anesthesia (peribulbar block), routine phacoemulsification complicated by a posterior capsular rent was first done with the implantation of a sulcus intraocular lens. Then, a standard 23-gauge three-port pars plana vitrectomy was performed to clear the vitreous hemorrhage, after which a mushroom-shaped mass measuring approximately 6 disc diameter in size (Figure 6) was seen protruding into the vitreous cavity inferior to the fovea (the base of which was 1 disc diameter inferior to the inferior vascular arcade). Micro scissors were used to obtain two biopsy specimens from the mass. After that, complete tumor transretinal endoresection using the vitrector was performed, during which endodiathermy was used frequently to stop bleeding from the mass to ensure adequate visibility at all times. Perfluorocarbon liquid was injected for foveal protection, drainage of subretinal fluid, and stabilization of the retina, while the edges of the resultant retinotomy were carefully cauterized using endodiathermy (Figure 7). This was followed by argon laser photocoagulation peripheral to the endodiathermy marks, placing three rows of laser photocoagulation around the retinotomy (Figure 8). Finally perfluorocarbon liquid/air exchange was performed and 1000cs silicone oil was injected as a tamponade.

Both solid biopsy specimens and diluted vitreous samples from the aspirated fluid were sent to the lab for histopathological examination. However, the pathologist report showed that there was not enough sample for adequate histopathological grading, with mainly melanocytes in the slide.

**Figure 9:**
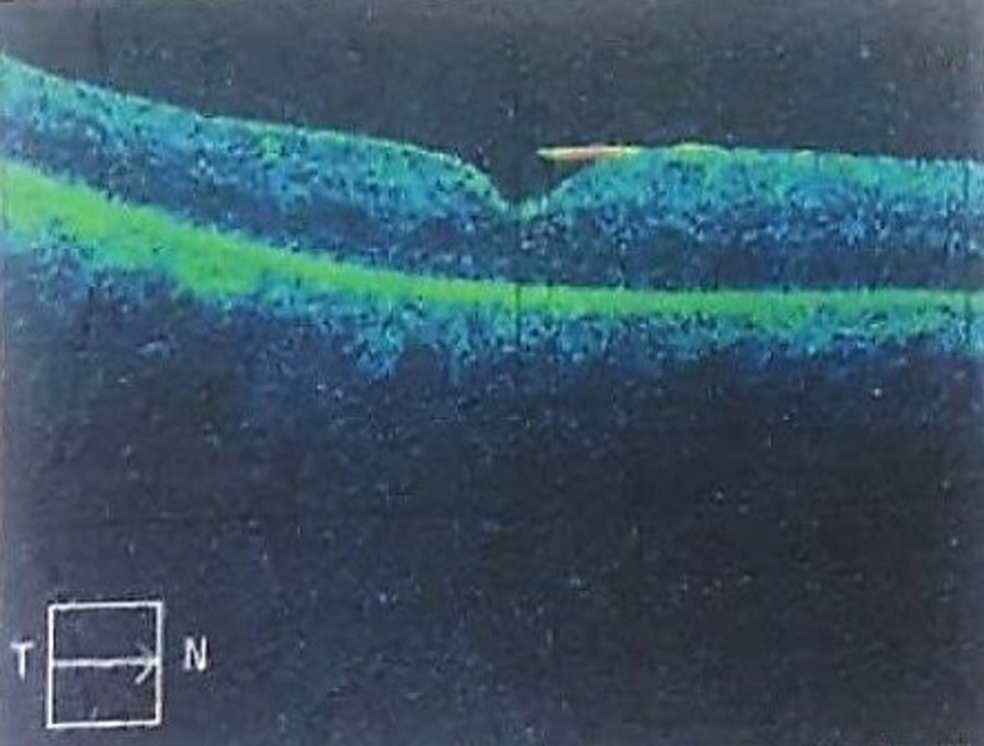
OCT macula at one month post-operative reveals normal foveal contour and absence of subretinal fluid. OCT, optical coherence tomography.

**Figure 10:**
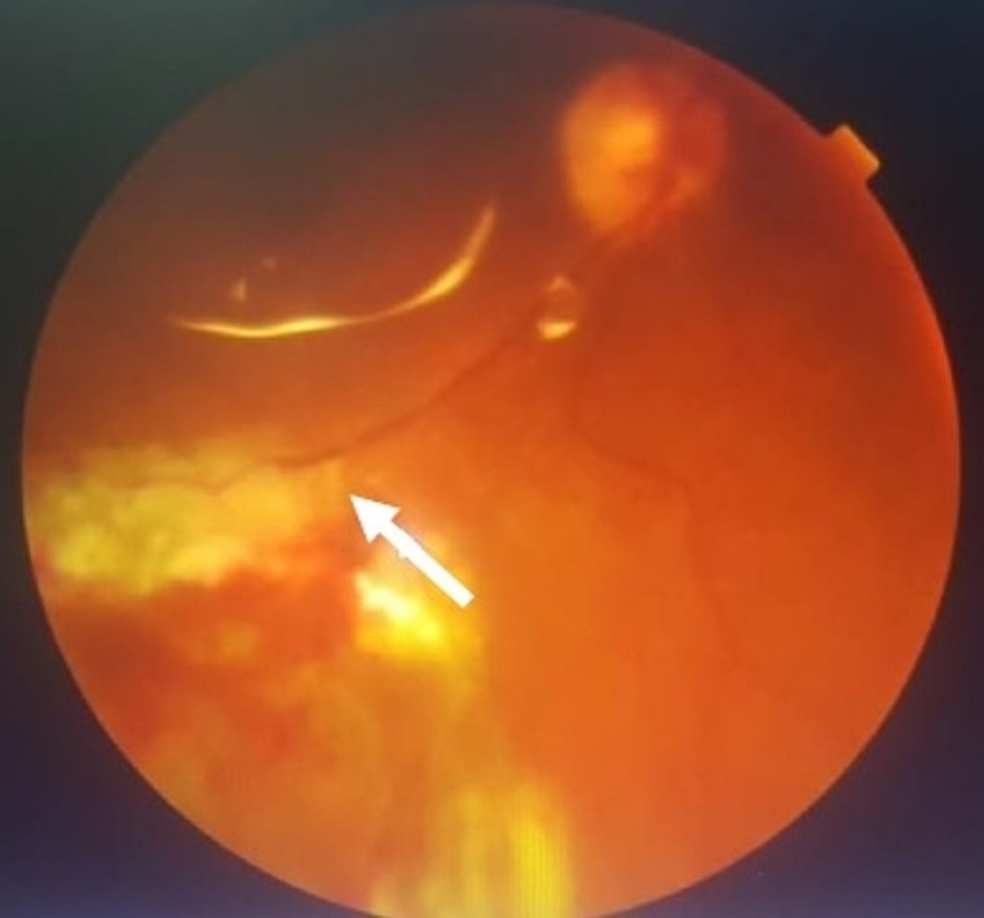
Colored fundus photograph of the right eye at one month post-operative. Photocoagulation scar (white arrow) after stabilization is still at the inferior arcade and does not affect the macula. Silicone oil reflection is noted.

At one week post-operation, the best-corrected visual acuity of the right eye had improved to 6/36 with remaining anterior uveitis and fundus examination showed a flat retina with white laser marks around the retinotomy. At one month post-operative, the visual acuity had further improved to 6/9 with a flat retina and silicone oil tamponade in situ. Optical coherence tomography (OCT) of the macula (Figure 9) and fundus photography (Figure 10) at one month post-operative are shown below.

**Figure 11:**
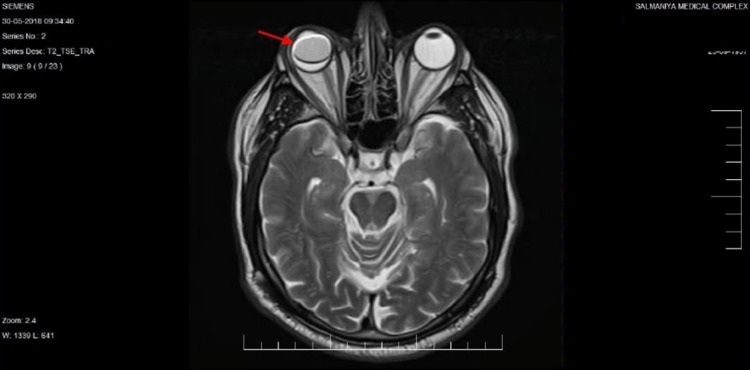
An axial T2-weighted MRI image of the orbits and brain done at one week post-operative shows no sign of the residual tumor, complete resolution of the exudative retinal detachment, and presence of silicone oil tamponade in the right eye (red arrow).

Post-operative MRI, which was done at one week post-operative to ensure complete resection, showed no evidence of residual tumor, and complete resolution of the exudative retinal detachment was seen pre-operatively, with a visible silicone oil bubble in the globe (Figure 11).

The patient was also referred to the oncology department to continue monitoring tumor control in liaison with the ophthalmology department with regular follow-up appointments, and he will be posted for silicone oil removal after six to nine months.

## Discussion

In this case report, we are sharing our experience of managing a solitary non-metastasized choroidal melanoma by pars plana endoresection, endodiathermy, and endolaser photocoagulation, which is, to the best of our knowledge, the first case of choroidal melanoma managed by pars plana endoresection to be reported in the Kingdom of Bahrain.

Globe preserving treatment modalities for localized choroidal melanoma such as brachytherapy have started to replace enucleation over the last decade [3,11]. However, radiation therapy is usually complicated by moderate to severe visual loss due to optic neuropathy, making it more suitable for patients with blind painful eyes, or those with poor visual prognosis. On the other hand, tumor endoresection was first reported by Damato et al. (1998) in an interventional case series, which showed excellent results in preventing local recurrences with preservation of central vision, making this approach an excellent choice for patients with a localized posteriorly located tumor, that is easily accessible via the pars plana route, yet far enough from the fovea and optic disc, a criterion that applies to the case mentioned in this report.

Patients’ expectations and lifestyles are also very important factors to consider in the treatment plan of such cases. Our patient was extremely alarmed by the visual loss that he suffered after developing vitreous hemorrhage, and despite retirement, he was still an active person establishing his private business and leading a socially active life. He clearly needed to maintain as good a vision as possible, as long as possible for him to maintain his quality of life. All these factors were thoroughly discussed with the patient when the treatment options were explained.

The most anticipated complications of tumor endoresection are cataract development and retinal detachment. Patients should be made aware of these complications and should understand the importance of continued follow-up. We managed to address the cataract at the time of surgery, because our patient had already had an early cataract in the affected eye, and phacoemulsification with intraocular lens implantation was done at the beginning of the surgery. Regular follow-ups with complete ophthalmic examinations will continue to be conducted to detect any recurrence of the subretinal fluid and/or retinal detachment.

Local disease control of choroidal melanoma is reportedly excellent regardless of the treatment modality chosen. However, up to 50% of patients ultimately develop metastatic disease despite treatment [3], which is why lifelong follow-up with interval MRI imaging, liver function tests, and other blood tests, in liaison with medical oncologists, are crucial in such cases.

## Conclusions

Endoresection can be an effective method for the management of solitary non-metastasized choroidal tumors, especially those that have become symptomatic. We presented this case to demonstrate how delicate endoresection via the pars plana route, combined with endodiathermy and endolaser, can be performed to successfully remove the tumor while preserving the patient’s central vision and the globe. This approach, though technically challenging, has proved to be useful when dealing with posteriorly located tumors that are easily accessible through the pars plana, and when the tumor is away from the fovea and optic nerve head. Compared to older modalities such as enucleation and radiotherapy, it has the advantage of preserving the globe and vision, which definitely has a huge impact on the patients' quality of life. However, lifelong follow-up is necessary to detect any signs of local recurrences timely.

## References

[1] Margo CE (2004). The collaborative ocular melanoma study: an overview. Cancer Control.

[2] Singh AD, Turell ME, Topham AK (2011). Uveal melanoma: trends in incidence, treatment, and survival. Ophthalmology.

[3] Krantz BA, Dave N, Komatsubara KM, Marr BP, Carvajal RD (2017). Uveal melanoma: epidemiology, etiology, and treatment of primary disease. Clin Ophthalmol.

[4] Damato EM, Damato BE (2012). Detection and time to treatment of uveal melanoma in the United Kingdom: an evaluation of 2,384 patients. Ophthalmology.

[5] Singh P, Singh A (2012). Choroidal melanoma. Oman J Ophthalmol.

[6] Kanski JJ, Bowling B, Nischal KK, Pearson A (2011). Clinical Ophthalmology: A Systematic Approach, 7th ed. Clinical Ophthalmology: A Systematic Approach, 7th ed.

[7] American Academy of Ophthalmology (2014). 2014-2015 Basic Clinical and Science Course. Section 4: Ophthalmic Pathology and Intraocular Tumors. Section 4: Ophthalmic Pathology and Intraocular Tumors.

[8] Triozzi PL, Singh AD (2014). Adjuvant therapy of uveal melanoma: current status. Ocul Oncol Pathol.

[9] Augsburger JJ, Corrêa ZM, Shaikh AH (2009). Effectiveness of treatments for metastatic uveal melanoma. Am J Ophthalmol.

[10] Postow MA, Kuk D, Bogatch K, Carvajal RD (2014). Assessment of overall survival from time of metastastasis in mucosal, uveal, and cutaneous melanoma. J Clin Oncol.

[11] Carvajal RD, Schwartz GK, Tezel T, Marr B, Francis JH, Nathan PD (2017). Metastatic disease from uveal melanoma: treatment options and future prospects. Br J Ophthalmol.

[12] Collaborative Ocular Melanoma Study Group (2006). The COMS randomized trial of iodine 125 brachytherapy for choroidal melanoma: V. Twelve-year mortality rates and prognostic factors: COMS report No. 28. Arch Ophthalmol.

[13] Caminal JM, Padrón-Pérez N, Arias L (2016). Transscleral resection without hypotensive anaesthesia vs iodine-125 plaque brachytherapy in the treatment of choroidal melanoma. Eye (Lond).

[14] Bechrakis NE, Bornfeld N, Zöller I, Foerster MH (2002). Iodine 125 plaque brachytherapy versus transscleral tumor resection in the treatment of large uveal melanomas. Ophthalmology.

[15] Vidoris AA, Maia A, Lowen M, Morales M, Isenberg J, Fernandes BF, Belfort RN (2017). Outcomes of primary endoresection for choroidal melanoma. Int J Retina Vitreous.

[16] Garcia-Arumi J, Leila M, Zapata MA, Velázquez D, Dinares-Fernandez MC, Tresserra F, Corcostegui B (2015). Endoresection technique with/without brachytherapy for management of high posterior choroidal melanoma: extended follow-up results. Retina.

